# Neuroprotective Effects of Danshen Chuanxiongqin Injection Against Ischemic Stroke: Metabolomic Insights by UHPLC-Q-Orbitrap HRMS Analysis

**DOI:** 10.3389/fmolb.2021.630291

**Published:** 2021-05-07

**Authors:** Peipei Zhou, Lin Zhou, Yingying Shi, Zhuolun Li, Liwei Liu, Lihua Zuo, Jun Zhang, Shuhong Liang, Jian Kang, Shuzhang Du, Jing Yang, Zhi Sun, Xiaojian Zhang

**Affiliations:** ^1^Pharmaceutical Department, The First Affiliated Hospital of Zhengzhou University, Zhengzhou, China; ^2^Precision Clinical Pharmacy Laboratory of Henan Province, Zhengzhou, China

**Keywords:** ischemic stroke, DSCXQ, UHPLC-Q-Orbitrap HRMS, metabolomics, anti-apoptotic

## Abstract

The incidence of cerebral ischemic stroke characterized by high mortality is increasing every year. Danshen Chuanxiongqin Injection (DSCXQ), a traditional Chinese medicine (TCM) preparation, is often applied to treat cerebral apoplexy and its related sequelae. However, there is a lack of systematic research on how DSCXQ mediates its protective effects against cerebral ischemia stroke. Metabolomic analysis based on UHPLC-Q-Orbitrap HRMS was employed to explore the potential mechanisms of DSCXQ on ischemic stroke induced by transient middle cerebral artery occlusion (MCAO). Pattern analysis and metabolomic profiling, combined by multivariate analysis disclosed that 55 differential metabolites were identified between Sham group and Model group, involving sphingolipid metabolism, glycerophospholipid metabolism, phenylalanine, tyrosine and tryptophan biosynthesis, primary bile acid biosynthesis, pantothenate and CoA synthesis and valine, leucine and isoleucine biosynthesis pathways. DSCXQ could reverse brain metabolic deviations in stroke by significantly upregulating the levels of L-tryptophan, Lyso (18:0/0:0), LPC (18:2), Indole-3-methyl acetate, and downregulating the levels of sphinganine 1-phosphate, L-threonic acid, glutaconic acid and N6,N6,N6-Trimethyl-L-lysine. In our study, we focused on the neuroprotective effects of DSCXQ against neuroinflammatory responses and neuronal apoptosis on a stroke model based on sphingolipid metabolism. The expressions of Sphk1, S1PR1, CD62P, Bcl-2, Bax, and cleaved Caspase-3 in brain tissue were evaluated. The neurological deficit, cerebral infarct size and behavioral abnormality were estimated. Results showed that DSCXQ intervention significantly reduced cerebral infarct size, ameliorated behavioral abnormality, inhibited the expression of Sphk1, S1PR1, CD62P, Bax, Cleaved Caspase-3, while increased the level of Bcl-2, and prevented neuronal apoptosis. The limitations are that our study mainly focused on the verification of sphingolipid metabolism pathway in stroke, and while other metabolic pathways left unverified. Our study indicates that SphK1-SIP axis may potentiate neuroinflammatory responses and mediate brain damage through neuronal apoptosis, and DSCXQ could suppress the activity of SphK1-SIP axis to protect brain tissue in cerebral ischemia. In conclusion, this study facilitates our understanding of metabolic changes in ischemia stroke and the underlying mechanisms related to the clinical application of DSCXQ.

## Introduction

Stroke is a dominant cause of long-term disability worldwide and the second most common cause of mortality after cardiovascular disease, bringing a massive socio-economic burden to the healthcare system and society (Feigin et al., 2016). At present tissue plasminogen activator (t-PA) remains the first choice of treatment in the clinic. However, a major limitation of r-tPA therapy is its narrow therapeutic window, which restrict the use of r-tPA as a long-term therapy (Fonarow et al., 2011). Although great progress has been made in understanding of the pathophysiology of stroke, a great portion of new drug trials have showed disappointing results (Jin et al., 2015; Hu et al., 2018), indicating that it is urgent to search for blood or brain biomarkers for stroke prognosis and new therapeutics.

Danshen Chuanxiongqin injection (CFDA approval # H52020959) is a TCM preparation composed of the extract of *Salvia miltiorrhiza Bunge* and *Conioselinum anthriscoides ‘Chuanxiong’*. The injection was listed in 2004 and is currently included in « Guidelines on the Rational Use of Chinese Drugs in Ischemic Stroke » as a recommended medication for the treatment of cerebral infarction and coronary heart disease. Mechanistic studies showed that DSCXQ could inhibit the production of malondialdehyde (MDA), effectively eliminating oxygen free radicals in rats and increasing the resistance of vascular endothelium to thrombosis (Fei et al., 2017; Zhang et al., 2020). However, previous studies did not fully explore the relevant mechanism of DSCXQ therapy in stroke at the level of metabolites. The mechanism of action of TCM drugs may be revealed by traditional pharmacological experiments to a certain extent, while combining metabolomic studies may help uncovering the overall metabolic network, making it a useful combination for discovering multiple interactions among the TCM components (Lyu et al., 2018). Herein we tried to fill such gap by scientifically studying the neuroprotective effects of DSCXQ on cerebral ischemia at metabolomics level. In order to systematically and scientifically study the neuroprotective effect of DSCXQ on cerebral ischemia from the perspective of multi-component, multi-target and multi-pathway, Metabolomics combined with pharmacological approaches were adopted to provide a novel way for the diagnosis and treatment of ischemic stroke.

In our previous study, A qualitative analytical method of ultraperformance liquid chromatography-quadrupole/orbitrap high resolution mass spectrometry (UHPLC-Q-Orbitrap HRMS) was established for identification and quantification of the constituents of Danshen-Chuanxiong Injection (see Supplementary Info 1) (Zhou et al., 2019b), which makes it possible to lay a solid foundation for our follow-up research. In the present study, ultra-high performance liquid chromatography coupled with a Q Exactive hybrid quadrupole-orbitrap high resolution mass spectrometry (UHPLC-Q-Orbitrap HRMS) was employed to profile metabolome of brain tissue in middle cerebral artery occlusion model rats and explore the intervention mechanism of DSCXQ. Immunohistochemistry, western blot, biochemical parameters, behavioral deficit and cognitive impairment were combined by metabolomics analysis to investigate the neuroprotective effects comprehensively, the potential biomarkers connecting with perturbed metabolic pathways were revealed. The limitations are that our study mainly focused on the verification of sphingolipid metabolism pathway in stroke, and while other metabolic pathways left unverified. This study unveil new insights in the understanding of pathological changes of stroke and the dynamic metabolomic profile with pharmacodynamic evaluation of DSCXQ based on metabolomics.

## Materials and Methods

### Chemicals and Reagents

Danshen Chuanxiongqin Injection was provided by the First Affiliated Hospital of Zhengzhou University. Chemical standards for MS/MS analysis of predicted metabolites were obtained from Sigma-Aldrich (St.Louis, MO, United States). HPLC grade acetonitrile and methanol were obtained from Fisher Scientific (Fair Lawn, NJ, United States). HPLC grade formic acid was purchased from Aladdin Industrial Co., Ltd. (Shanghai, China). Ultra-pure water (18.2 M) was prepared daily by a Milli-Q water purification system (Millipore, Shanghai, China). All solutions were filtrated by 0.22 μm pore size filters before use.

### Cerebral Ischemia/Reperfusion Model and Drug Administration

Adult male Sprague-Dawley rats (weighing 220-250 g) were obtained from experimental animal center of Zhengzhou university (Zhengzhou, China). Animals were fed with free water and diet under the controlled temperature (25 ± 2°C), humidity (60 ± 5%) and 12 h light/12 h dark cycle for 7 days to adapt to the environment. Rats were fasted overnight before the surgical operation, all experiments were carried out in adherence with the standard guidelines for the Care and Use of laboratory animals from the National Institute of Health (NIH) and handled strictly according to obligations of the Animals Ethics Committee of Zheng Zhou University.

The MCAO rat model was induced by the intraluminal technique according to the method of originally described by Longa et al. (1989), with little modification. Briefly, the right common carotid artery (CCA), external carotid artery (ECA), and internal carotid artery (ICA) were isolated clearly and exposed with caution on rats which were anesthetized with 3% chloral hydrate (1 ml/100 g) intraperitoneally, then a poly nylon monofilament of 0.24 mm in diameter with tip rounded was inserted through ECA into the ICA to block the origin of MCA until the slightly resistance was felt, nearly 18-20 mm. rats were achieved 2 h cerebral ischemia and then pulled out the filament to complete 24 h reperfusion. Meanwhile, the temperature of rats were kept at 36.5–37.5°C with a thermostat-controlled heating pad. The sham operation was performed the same surgical procedures except for inserting a filament.

Rats were randomly divided into three groups with six in each: sham-operated (sham), model (MCAO) and DSCXQ-treated groups, During the whole experiments process, the research group carried out three batches of animal experiments. Rats in Model and DSCXQ group received ischemia-reperfusion (I/R) surgery, while rats of the Sham group underwent artery exposure and isolation without the insertion. DSCXQ was intravenously administered to rats for 7 days and rats in the Sham and Model groups were intravenously administered with the correspond volume of 0.9% saline in the same way. DSCXQ injection is a mixture of TCM compounds which contains extracts from salvia miltiorrhiza (200 mg/ml) and ligustrazine (see Supplementary Info 1). Rats were administered intravenously with DSCXQ (0.16 ml/100 g of body weight) once a day starting immediately post surgery.

### Brain Sample Collection and Preparation

After 2 h of ischemia followed by 24 h reperfusion, rats were sacrificed and brains were quickly removed. Samples were collected with the method that 0.5 g brain tissue was homogenized with triple volume saline (w/v), and centrifuged at 3,000 rmp for 10 min, then the supernatant were separated and stored at −80°C for reserve. Sample of 100 ml was transferred into 1.5 ml EP tube and added with 300 μl methanol containing 500 ng/ml ketoprofen and 50 ng/ml 2-chloro-Lphenylalanine as internal standard. After vortexing for 3 min, the mixture was centrifuged at 13,000 rpm for 10 min at 4°C. Lastly, 200 ml of the supernatant was transferred to an autosampler vial to UHPLC-Q-Orbitrap HRMS for metabolomics analysis. To evaluate stability and accuracy of the UPLC-MS/MS analysis system, 20 μl of samples from each group were mixed and generated the pooled quality control (QC) samples which were used the same method to analyze companying with measured samples. Six QC samples were inserted every five injections during the whole measurement process for quality control.

### UHPLC-MS/MS System Conditions

#### Chromatography

Chromatographic experiments were performed on Dionex Ultimate 3000 UHPLC system (Thermo Fisher Scientific, San Jose, CA, United States). An aliquot of 5 μL samples was injected into BEH C18 (2.1 × 100 mm, 1.7 μm) maintained at 4°C and the flow rate was 0.35 mL/min, the mobile phase was formed of solvent A (0.1% formic acid-water, V/V) and solvent B (acetonitrile), the gradient elution was as follows: 0-1 min, 95% A; 1-12 min, 0% A; 12-15 min, 95% A.

#### Mass Spectrometry

The MS spectrometry was performed on a Q-Orbitrap mass spectrometer with high resolution (Thermo Scientific, San Jose, United States) using a heat electrospray ionization (HESI) ion source, the main parameters were set as follows: capillary temperature of 320°C, Aux gas flow rate of 10 Arb, spray voltage of 3.5 kV (ESI^+^)/2.8 kV (ESI^–^), sheath gas flow rate of 40 Arb (+)/38 Arb (−), the full scan data acquired ranges from 80-1200 m/z with a resolution of 70,000 and a resolution of 17,500 resolution in MS^2^ mode, samples were analyzed at 20, 30, 40 NCE (normalized collisional energy).

### Evaluation of Neurological Deficit

Neurobehavioral dysfunction of rats was scored and evaluated by one investigator who was blinded to the experimental design. Neurological deficits were estimated by Zea Longa five-point scale as follows: 0 points indicated no neurobehavioral dysfunction; 1 points showed the nerve function injuries as left forepaw failed to extend fully which represented a mild focal neurologic deficit; 2 points suggested a moderate focal neurologic deficit accompanied by circling to the left; and a score of 3 meant a severe focal deficit as rats were fallen to the affected side and crawled slowly; 4 points represented rats did not walk spontaneously and had a depressed level of consciousness. The inclusion criterion of the model was a neurological function score of 1–3, and excluded a neurological function score of 0 or 4.

### Measurement of Cerebral Infract Area

Cerebral infarct area was measured by 2,3,5-triphenyltetrazolium chloride (TTC) staining. TTC staining is conventional used for visualization of hypoxic brain tissue the fast and reliably and for measuring the size of cerebral infarction. After evaluating the neurological function score, rats were sacrificed using the approved protocol. The brain tissues of rats were removed and frozen at −20°C for 20 min, then sectioned into five slices (2 mm thick) along the coronal plane, after that the brain slices were stained with 2% TTC dissolved with 0.9% saline for 20 min at 37°C in the dark, following fixed in 4% paraformaldehyde overnight. Normal cerebral tissue was stained (red) whereas the infarct tissue unstained (white). The infracted tissue areas were analyzed by weighing the infarct area, and infarct area content was calculated as infarct weight/(infarct area + normal area weight) × 100%. The infarct areas were statistically analyzed as percentages of the total slice areas.

### Immunohistochemical Determination of CD62P

Paraffin-embedded sections were used to assess the expression of CD62P according to standard histological procedures. The brain tissues were conventionally fixed in 4% paraformaldehyde and embedded in paraffin wax. The samples were serially sectioned at 4 μm by a tissue-slicing machine. After dewaxing and hydration. The blocking of endogenous peroxidases was achieved by incubating the sections in 3% hydrogen peroxide. After that, the tissue antigen was repaired by microwave, and the blocking solution containing goat serum incubated on sections at 37°C for 20 min. Then Rabbit Anti-CD62P antibody (Abcam, Cambridge, United Kingdom) was added to the sections and incubated overnight at 4°C. Wash slides three times and remove excess liquid from around the sections, goat antirabbit immunoglobulin G (IgG) antibody (Proteintech Group Inc., Wuhan, China) labeled with horseradish peroxidase was added to the sections and incubated at 37°C for 30 min. After reacted with DAB solution, the stained tissue sections were observed under a 200 × light microscope in three visual fields of the ischemic cortex region of the infarct.

### TUNEL Staining

TUNEL staining was employed to estimate cell apoptosis following standard’s instructions. Brain tissues were fixed in 10% formaldehyde in PBS for 24 h and embedded in paraffin blocks. The blocks were cut into 4 μm thickness, by heating the slides for 10 min at 70°C and followed by two 5-min incubations in a fresh xylene bath at room temperature in a dyeing jars. Then the tissue samples were transferred through a graded ethanol series to rehydrate. Nuclear proteins were stripped from the DNA by incubation 20 mg/mL of proteinase K solution for 30 min, and endogenous peroxidase was inactivated with 2% H_2_O_2_ for 5 min at room temperature. Sections were incubated in a buffer containing TdT in a humidified chamber for 30 min at 37°C. and digoxigenin labeled dUTP followed by digoxigenin-conjugated peroxidase treatment. Stop the reaction by incubating the slides in 2 × SSC for 15min. Washed the slides and incubated in Streptavidin HRP diluted with PBS at the ratio of 1:500 for 30 min at 37°C, DAB (diaminobenzidine) was used as the chromogen. The TUNEL-positive cells stained brown granules were considered apoptotic due to the binding of dUTP enzyme to 3′- OH terminal of broken DNA, which were observed under a 200 × light microscope in three visual fields of the ischemic cortex region.

### Western Blotting Analysis

Western blotting analysis were performed according to our previous study (Zhou et al., 2019a). In brief, the total proteins of brains were extracted by ice cold cell lysis buffer and determined the total content by Bio-Rad DC Protein Assay Kit (BIO-RAD, China). Equal quantities of protein samples (40 μg) were loaded into SDS-PAGE and transferred to PVDF membranes. Membranes were probed with primary antibodies against Sphk1 (1:1000), S1PR1 (1:1000) (Affinity Biosciences, OH, United States), Bcl-2, Bax (1:1000), Cleaved Caspase-3 (1:500) (Cell Signaling Technology, Boston, MA, United States) at 4°C overnight. Subsequently, the membrane was washed with Tris-buffer saline containing 0.05% Tween 20 (TBST) buffer three times and probed with secondary antibody conjugated horseradish peroxidase. Protein visualization was achieved by the enhanced chemiluminescence reagents on a gel imaging system (Tannon-5200, Shanghai, China).

### Data Processing and Statistical Analysis

The data was acquired and processed by Thermo XcaliburTM software (Version 3.0, Thermo Scientific), and Thermo Scientific Compound Discoverer 3.0 software was employed to pretreat LC-MS raw data. The spectra were chosen from LC-MS data files and retention time alignment was achieved according to mass tolerance and time shift criteria. Preliminary identification of metabolites was fulfilled by searching databases containing ChemSpider, Mass Lists, mzCloud, mzVault, and local database. Then the result data matrix were input into software SIMCA (version 14.0, Umetrics, Umea, Sweden) for multivariate statistical analysis, including the subsequent principal component analysis (PCA) and orthogonal partial least square discrimination analysis (OPLS-DA), The metabolites with variable importance in the projection (VIP) values >1.0 and *p* values <0.05 for Model versus Sham were screened as potential biomarkers of MCAO. In addition, student’s *t*-test and fold change value were also applied to further screen out the significant variables between different groups.

Quantitative data were expressed as mean ± standard deviation (S.D.) using statistical software SPSS19.0. Differences were assessed by one-way analysis of variance (ANOVA) followed by a least significant difference t-test (LSD). Differences were considered significant at *p* < 0.05.

## Results

### Assessment of QC Samples

**FIGURE 1:**
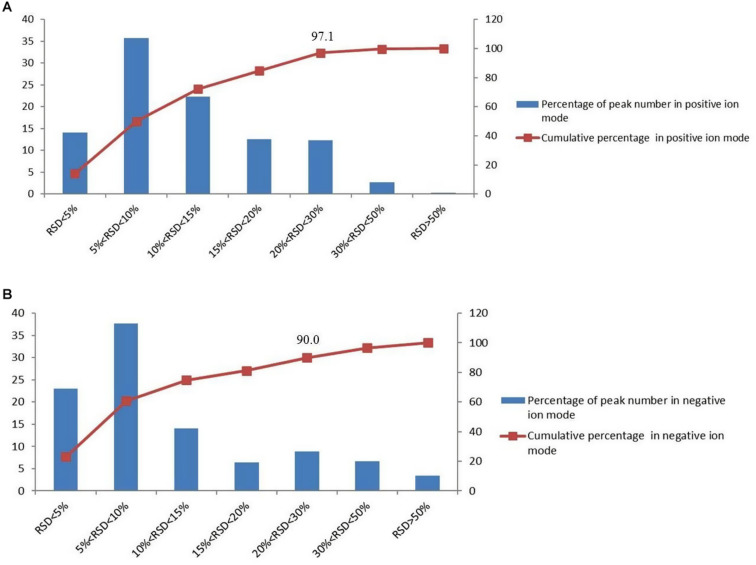
The distributions of the coefficient of variation for each metabolite in QC samples, **(A)** percentage (%) of all detected peaks in positive mode; **(B)** percentage (%) of all detected peaks in negative mode.

QC samples are used to verify the stability of the experiment. In the process of metabolomics, QC samples were inserted every five injections during the whole measurement process. And the relative standard deviation (RSD) of the peak area in the chromatogram was used to calculate to evaluate the method performance. In the negative and positive mode, more than 90% of RSD is lower than 30%, which indicates that the analysis system is stable and reliable (Figure 1). Therefore, the differences of metabolic markers found in this study can truly reflect the differences of biological status among sample groups.

### Multivariate Data Analysis for Brain Samples

**FIGURE 2:**
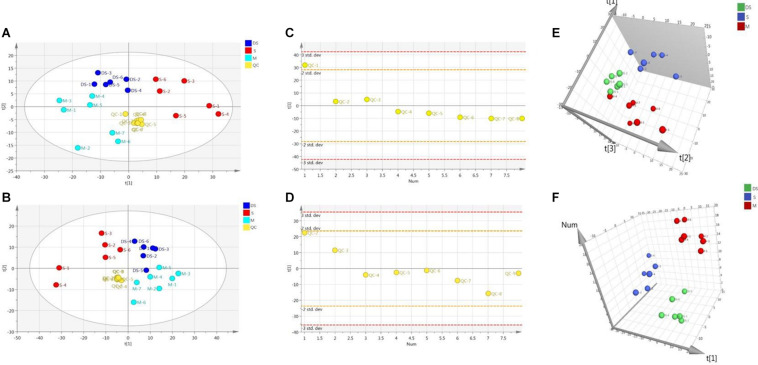
**(A)** The PCA plot of samples and QC in positive ion mode, **(B)** The PCA plot of samples and QC in negative ion mode, **(C)** The scatter distribution plot in the first principal components in positive ion mode, **(D)** The scatter distribution plot in the first principal components in negative ion mode. (Note: QC represents quality control, S represents Sham group, M represents Model group, DS represents DSCXQ group), **(E)** 3-D PLS-DA score plot of the different groups in positive ion mode, **(F)** 3-D PLS-DA score plot of the different groups in negative ion mode.

**FIGURE 3:**
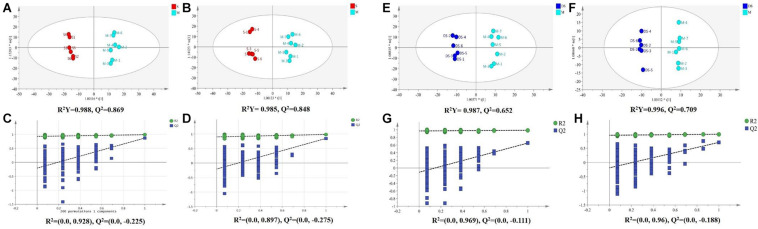
**(A)** The OPLS-DA plot between Sham and Model group in positive ion mode, **(B)** The OPLS-DA plot between Sham and Model group in negative ion mode, **(C)** The permutations test of Sham vs. Model groups in positive ion mode, **(D)** The permutations test of Sham vs. Model groups in negative ion mode; **(E)** The OPLS-DA plot between Model and DSCXQ group in positive ion mode, **(F)** The OPLS-DA plot between Model and DSCXQ group in negative ion mode. **(G)** The permutations test of Model vs. DSCXQ groups in positive ion mode, **(H)** The permutations test of Model vs. DSCXQ groups in negative ion mode, (Note: S represents Sham group, M represents Model group, DS represents DSCXQ group).

Based on UPLC-MS/MS, the brain samples of rats were analyzed in positive and negative ion mode. In order to investigate the overall changes of metabolites in rats with cerebral ischemia-reperfusion, PCA method was used to identify the metabolites spectrum data of rats in Sham group and Model group, moreover the sample distribution map which reflect the degree of similarity and difference between samples was obtained. In the map, samples with small difference in atlas are close to each other, on the contrary, samples with large difference are far apart. In PCA model, Sham group, Model group and DSCXQ group are apparent separated on the scatter plot after automatic fitting (Figure 2), to further amplify the differences between groups, the supervised multidimensional analysis method OPLS-DA was adopted (see Figure 3). Sham group, Model group and DSCXQ group were well distinguished in PCA, Figures 3A,B shows that the Model group and Sham-operated group can be distinguished obviously under the positive and negative ion mode, which indicates the model was successful. Compared with the Sham group, the metabolites in Model group change obviously and abnormal. Significant distinctions were acquired with R^2^Y at 0.988 and Q^2^ at 0.869for ESI^+^ mode (Figure 3A), and R^2^Y at 0.985 and Q^2^ at 0.848 for ESI^–^ mode (Figure 3B), meanwhile, the OPLS-DA model is validated by a permutation of 200 times and the results (R^2^ = 0.928, Q^2^ = −0.225 for ESI^+^ mode; R^2^ = 0.897, Q^2^ = −0.275 for ESI^–^ mode). The OPLS-DA model between DSCXQ and Model group were performed to display the neuroprotection of DSCXQ vs. Model group. Notable differentiation was obtained with R^2^Y at 0.987and Q^2^ at 0.652 for ESI^+^ mode (Figure 3E), and R^2^Y at 0.996 and Q^2^ at 0.709 for ESI^–^ mode (Figure 3F). To assess the validity of the OPLS-DA model, permutation test with 200 measurements was performed and the result (R^2^ = 0.969, Q^2^ = −0.111 for ESI^+^ mode; R^2^ = 0.96, Q^2^ = −0.188 for ESI^–^ mode) indicated that there was no overfitting of the model.

### Biomarkers Identification

**FIGURE 4:**
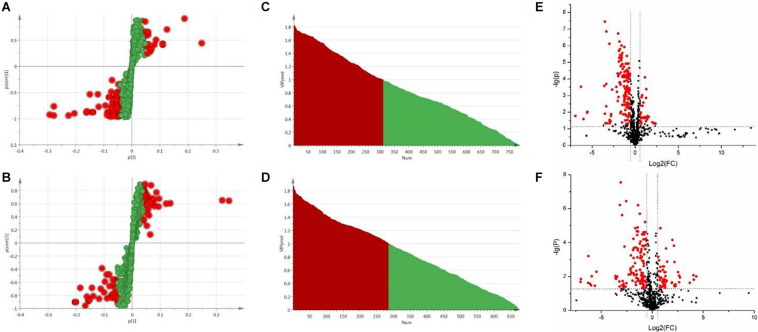
**(A)** The S-plot of Sham vs. Model groups in positive ion mode, **(B)** The S-plot of Sham vs. Model groups in negative ion mode, **(C)** The VIP plot of Sham versus Model groups in positive ion mode, **(D)** The VIP plot of Sham vs. Model groups in negative ion mode, **(E)** The volcano plot of Sham vs. Model groups in positive ion mode, **(F)** The volcano plot of Sham vs. Model groups in negative ion mode.

In OPLS-DA analysis, each point in S-plot graph of Sham group, Model group and DSCXQ group represents a variable, and the importance of each point for classification is measured by their values, screened according to VIP (variable importance in the projection, VIP). VIP >1 is considered to be a significant variable contributing to the model. The farther away from the center, the greater the contribution of variables to the difference and the more likely they are to become potential characteristic metabolites. Metabolites distributed far from the origin play an important role in the S-plot graph (see Figures 4A,B). In addition, the t test and folding changes of the students were calculated to ensure that the metabolites detected were significantly changed in concentration. *p* ≤ 0.05 and fold change ≥1.5 (or fold change ≤0.67) were retained in order to obtain more credible and distinct markers. In volcanic maps, the red dots indicate that the *p* values of metabolites are less than 0.05 and fold change ≥1.5 or fold change ≤0.67 (log_2_FC ≥ 0.58 or log_2_FC ≤ −0.58), which help to determine the potential biomarkers of stroke.

The chemical structures of reserved metabolites were identified by searching accurate molecular mass data and MS/MS fragments in database such as HMDB, METLIN, M/Z cloud, and the database established by ourselves. Finally, in all 55 potential biomarkers that distinguish the difference between the Sham group and Model group were identified in positive and negative mode (Table 1). Of the potential biomarkers, it can be revealed that lipids metabolic perturbations play an important role in Model group, including the increased sphingolipids, declined lysophosphatidyl cholines (LysoPCs), lysophosphatidylethanolamines (LysoPEs) and bile acids. Secondly, it is amino acid metabolism, containing the decreased branched amino acids and the imbalance of arginine-NO cycle observed in Model group (shown in Table 1).

**TABLE 1:** Identified endogenous metabolites between Sham group vs. Model group and adjusted in DSCXQ group in rats brain.

No	m/z	Metabolites	Formula	RT (min)	VIP	Fold change (M/S)	Fold change (DS/M)	Pathway Involved	ppm
1	118.086	L-Valine^#^	C_5_H_11_NO_2_	0.84	1.699	0.567	1.067 ↑	Valine, leucine and isoleucine biosynthesis	–3.431
2	147.125	Acetylcholine^#^	C_7_H_16_NO_2_	0.828	1.374	1.930	0.747 ↓	Glycerophospholipid metabolism	–2.705
3	153.066	N1-Methyl-2-pyridone-5-carboxamide	C_7_H_8_N_2_O_2_	1.179	1.579	0.308	0.473	Nicotinate and nicotinamide metabolism	–3.489
4	175.118	D-Arginine^#^	C_6_H_14_N_4_O_2_	0.756	1.361	1.504	0.822 ↓	D-Arginine and D-ornithine metabolism	–2.868
5	176.103	Citrulline^#^	C_6_H_13_N_3_O_3_	0.824	1.551	0.649	0.836	Arginine and proline metabolism	–2.713
6	182.081	L-Tyrosine^#^	C_9_H_11_NO_3_	1.104	1.559	0.481	1.058 ↑	Phenylalanine, tyrosine and tryptophan biosynthesis	–0.438
7	188.071	Indoleacrylic acid	C_11_H_9_NO_2_	5.113	1.407	0.248	4.073 ↑**	tryptophan metabolism	–1.144
8	189.160	N6,N6,N6-Trimethyl-L-lysine^#^	C_9_H_20_N_2_O_2_	0.76	1.597	2.473	0.586 ↓**	Lysine degradation	–1.609
9	204.123	L-Acetylcarnitine^#^	C_9_H_17_NO_4_	1.076	1.581	0.487	1.010 ↑	Beta Oxidation of Very Long Chain Fatty Acids	–1.737
10	205.097	L-Tryptophan^#^	C_11_H_12_N_2_O_2_	3.042	1.680	0.541	1.256 ↑*	Tryptophan metabolism	–1.044
11	218.138	Propionylcarnitine	C_10_H_19_NO_4_	1.328	1.102	1.600	0.767 ↓	Oxidation of Branched Chain Fatty Acids	–1.305
12	220.118	Pantothenic acid^#^	C_9_H_17_NO_5_	2.269	1.245	2.159	0.762 ↓	Pantothenate and CoA biosynthesis	–1.041
13	232.154	Butyrylcarnitine	C_11_H_21_NO_4_	2.742	1.058	1.638	0.723 ↓	Synthesis and degradation of ketone bodies	–1.355
14	246.170	2-Methylbutyro ylcarnitine	C_12_H_23_NO_4_	3.534	1.065	1.642	0.543 ↓*	Synthesis and degradation of ketone bodies	–1.725
15	248.149	(R)-3-hydroxybutyryl carnitine	C_11_H_21_NO_5_	1.075	1.225	0.491	0.530	Synthesis and degradation of ketone bodies	–1.931
16	302.305	Sphinganine^#^	C_18_H_39_NO_2_	7.434	1.141	2.404	0.764 ↓	Sphingolipid metabolism	–2.402
17	450.321	Chenodeoxycholic acid^#^	C_26_H_4__3_NO_5_	6.767	1.253	0.008	1.393 ↑	Primary bile acid biosynthesis	–1.428
18	466.316	Glycocholic acid	C_26_H_43_NO_6_	5.926	1.615	0.013	2.956 ↑	Primary bile acid biosynthesis	–1.155
19	468.308	LysoPC(14:0/0:0)	C_22_H_46_NO_7_P	7.515	1.361	0.591	1.244 ↑	Arachidonic Acid Metabolism	–2.468
20	480.308	LysoPE(18:1/0:0)	C_23_H_46_NO_7_P	8.593	1.498	0.602	0.972	Glycerophospholipids Metabolism	–2.219
21	482.324	LysoPE(18:0/0:0)	C_23_H_48_NO_7_P	7.942	1.782	0.332	1.343 ↑*	Glycerophospholipids Metabolism	–2.313
22	497.347	1-palmitoylglycero phosphocholine	C_24_H_51_NO_7_P	8.203	1.382	0.646	1.030 ↑	Phospholipid metabolism	–1.865
23	508.376	LysoPC(P-18:0)	C_26_H_54_NO_6_P	8.774	1.675	0.407	1.098 ↑	Glycerophospholipids Metabolism	–0.829
24	510.355	LysoPC(17:0)	C_25_H_52_NO_7_P	8.811	1.436	0.442	0.993	Glycerophospholipid metabolism	–0.247
25	520.340	LPC(18:2)	C_26_H_50_NO_7_P	8.004	1.717	0.538	1.378 ↑	Glycerophospholipid metabolism	–1.952
26	522.355	LysoPC(18:1)	C_26_H_52_NO_7_P	8.56	1.559	0.534	1.061 ↑	Glycerophospholipid metabolism	–1.887
27	570.355	LysoPC(22:5)	C_30_H_52_NO_7_P	8.372	1.062	0.350	1.075 ↑	Glycerophospholipid metabolism	1.375
28	572.371	LysoPC(22:4)	C_30_H_54_NO_7_P	8.589	1.409	0.359	1.287 ↑	Glycerophospholipid metabolism	–2.194
29	703.575	Palmitoyl sphingomyelin	C_39_H_79_N_2_O_6_P	7.987	1.708	0.614	1.001↑	Sphingolipids metabolism	–2.375
30	324.964	Trichloroethanol glucuronide	C_8_H_11_C_l__3_O_7_	3.895	1.215	2.241	0.586 ↓	Glucuronic Acid Derivatives	4.173
31	105.055	3-Hydroxybutyric acid^#^	C_4_H_8_O_3_	1.236	1.439	0.340	0.541	Ketone metabolism	7.855
32	131.070	2-Methyl-3-ketovaleric acid	C_6_H_10_O_3_	3.714	1.556	0.469	1.157 ↑	leucine metabolism	6.659
33	393.300	Deoxycholic acid^#^	C_24_H_40_O_4_	7.57	1.118	0.143	0.756	bile acid metabolism	3.000
34	407.279	3,7-Dihydroxy-12-oxocholanoic acid	C_24_H_38_O_5_	6.437	1.073	0.139	0.408	bile acid metabolism	2.022
35	135.029	Malic acid^#^	C_4_H_6_O_5_	0.843	1.169	0.443	0.884	TCA cycle	6.242
36	193.034	Citric acid^#^	C_6_H_8_O_7_	0.837	1.080	0.500	0.665	Citrate cycle (TCA cycle)	5.659
37	117.054	Levulinic acid	C_5_H_8_O_3_	1.849	1.532	0.507	1.070 ↑	Gamma-ketoacid and derivatives	6.601
38	119.070	2-Hydroxyvaleric acid	C_5_H_10_O_3_	2.927	1.747	3.927	1.070 ↓	fatty acid metabolism	7.085
39	199.057	4-Methyldiben zothiophene	C_13_H_10_S	3.484	1.704	0.450	1.292 ↑	Cell membrane	6.102
40	131.034	Glutaconic acid	C_5_H_6_O_4_	0.836	1.197	1.843	0.520 ↓*	glutamic acid metabolism	5.696
41	137.044	L-threonic Acid	C_4_H_8_O_5_	0.83	1.364	1.688	0.617 ↓*	amino acid metabolism	6.148
42	450.321	Glycoursode oxycholic acid	C_26_H_43_NO_5_	5.921	1.271	0.008	1.236 ↑	Primary bile acid biosynthesis	2.387
43	500.304	Taurochenodes oxycholic acid	C_26_H_45_NO_6_S	5.768	1.423	0.349	1.119 ↑	Primary bile acid biosynthesis	1.414
44	133.086	2-Hydroxycaproic acid	C_6_H_12_O_3_	3.941	1.589	2.760	1.420	Fatty acid metabolism	5.869
45	205.097	Tryptophan^#^	C_11_H_12_N_2_O_2_	3.039	1.518	0.548	1.218 ↑	Aminoacyl-tRNA biosynthesis	5.839
46	466.316	Glycocholic acid	C_26_H_43_NO_6_	5.936	1.586	0.013	3.001 ↑	Primary bile acid biosynthesis	1.821
47	220.118	Pantothenic acid^#^	C_9_H_17_NO_5_	2.256	1.105	1.998	0.658 ↓	CoA synthesis	4.177
48	516.299	Taurocholic acid	C_26_H_45_NO_7_S	5.881	1.638	0.322	1.610 ↑	Taurine and hypotaurine metabolism	1.478
49	190.086	Indole-3-methyl acetate	C_11_H_11_NO_2_	5.32	1.575	0.098	4.223 ↑**	tryptophan catabolism	–1.500
50	175.096	Suberic acid	C_8_H_14_O_4_	4.265	1.164	0.261	1.009 ↑	Fatty acid metabolism	4.418
51	382.272	Sphinganine 1-phosphate	C_18_H_40_NO_5_P	7.636	1.657	2.561	0.532 ↓**	Sphingolipid Metabolism	2.981
52	167.070	D-Phenyllactic acid	C_9_H_10_O_3_	4.287	1.239	2.360	0.765 ↓	amino acid metabolism	4.661
53	160.097	Isovalerylglycine	C_7_H_13_NO_3_	3.284	1.273	0.466	0.794	carboxylic acids and derivatives	4.556
54	208.097	N-Acetyl-L-phenylalanine	C_11_H_13_NO_3_	4.315	1.300	2.549	0.815 ↓	phenylalanine metabolism	5.436
55	305.247	Arachidonic acid	C_20_H_32_O_2_	7.874	1.619	0.574	0.890	Arachidonic acid metabolism	3.375

^#^metabolites were identified by reference standards; *p values < 0.05; **p values <0.01; RT, retention time; VIP, variable importance in the projection obtained from Model group vs. Sham group.

### Metabolic Pathway Analysis

**FIGURE 5:**
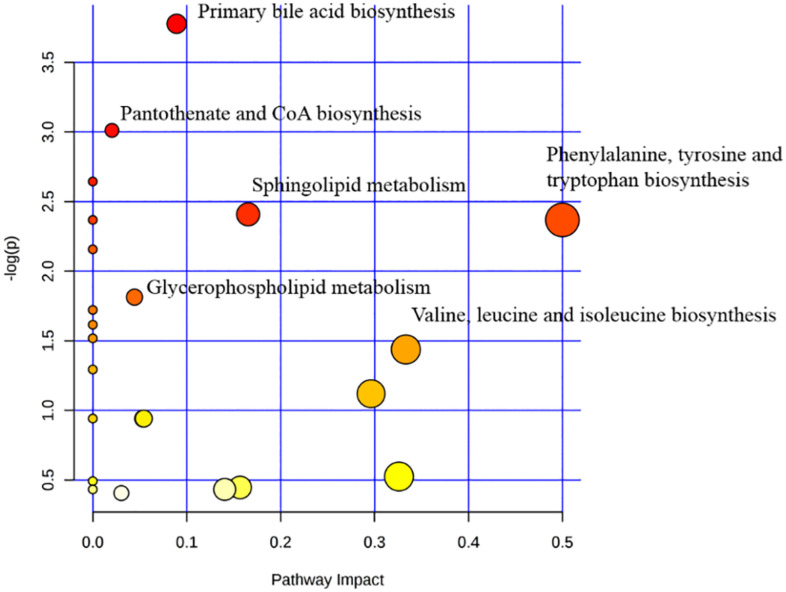
The pathway analysis of the identified metabolites.

**FIGURE 6:**
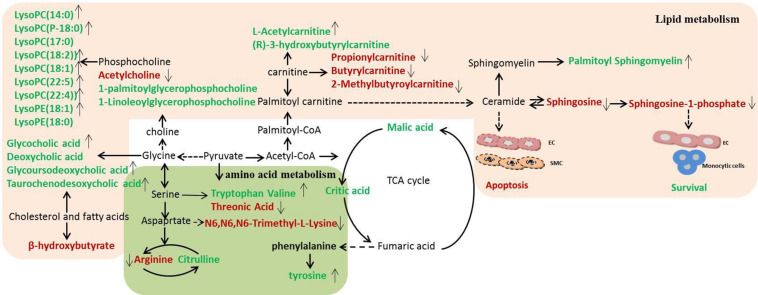
Disordered metabolic pathway network in stroke and the interventional effects of DSCXQ. The names marked in red represent up-regulated metabolites in model rats, and the names marked in green represent down-regulated metabolites in model rats. The names marked in black represent undetected metabolites. The metabolites reversed by DSCXQ are marked with up and down arrows.

In order to clarify the pathogenesis of stroke, the metabolites were input into MetaboAnalyst 3.0 to construct metabolic pathways discovering the important pathways (Figure 5). And a metabolic correlation network (Figure 6) was established by searching online database KEGG^1^ and HMDB^2^. Metabolic dysregulations in rats with cerebral ischemia induced by MCAO are mainly related to six metabolic pathways, which involved in sphingolipid metabolism, glycerophospholipid metabolism, phenylalanine, tyrosine and tryptophan biosynthesis, primary bile acid biosynthesis, pantothenate and CoA synthesis and valine, leucine and isoleucine biosynthesis.

In the pathway of phenylalanine, tyrosine and tryptophan biosynthesis, the levels of tyrosine, the metabolite of phenylalanine, decreased in Model compared to Sham group, which suggested the emergence of excitotoxicity induced by glutamate in cerebral ischemia (Kagiyama et al., 2004). A high level of N6,N6,N6-Trimethyl-L-lysine and threonine might correlate with disruption of the nervous system, for the metabolism of glycine, serine and threonine play an essential role in the function of the central nervous system (Tabatabaie et al., 2010; Amelio et al., 2014). The down-regulated tryptophan indicates that apoptosis mechanism was induced in Model group, and studies have revealed that the active degradation of kyn pathway in Trp were closely linked to the severity and long-term prognosis of stroke (Wang et al., 2015). In the pathway of valine, leucine and isoleucine biosynthesis, it was found that neurological dysfunction went hand in hand with the down regulation of catabolism in BCAAs(branched-chain amino acid, BACC), and decreased valine with stroke might be attributed to either an inverse feedback mechanism or the use as an energy source (Kimberly et al., 2013). Lipid metabolites are crucial to the progression of stroke and are likely to be a potential target of interventions for stroke. The levels of lipid-related metabolites, e.g., LysoPCs, LysoPEs, sphinganine, Sphinganine 1-phosphate and fatty acids were significantly altered in Model group. Decreased LysoPCs and LysoPEs levels maybe the inhibition of phospholipase A2 activity by LPCs *in vivo* (Cunningham et al., 2008). While elevated sphinganine and Sphinganine 1-phosphate were closely related to dysregulation of inflammatory responses and apoptosis (Maceyka et al., 2012). The altered levels of 3-hydroxybutyrylcarnitine, propionylcarnitine, Butyrylcarnitine and 2-Methylbutyroylcarnitine, indicated the metabolic dysregulations of fatty acid β oxidation and oxidative stress (Virmani and Binienda, 2004). Other metabolites are mostly basic organic acids from many basal metabolic pathways, such as citric acid, malic acid, 3-hydroxybutyric acid, most of which were TCA pathway intermediates (Gao et al., 2008). In addition, the decreased of bile acids, e.g., glycocholic acid, deoxycholic acid, glycoursodeoxycholic acid, and taurochenodesoxycholic acid, were observed in stroke rats, which implicated the gut microbiome was involved in the progression of stroke (Kasahara and Rey, 2019).

After intervened by DSCXQ, the disorder of metabolites has been improved. DSCXQ reversed brain metabolic deviations in stroke by significantly upregulating the levels of L-tryptophan, Lyso (18:0/0:0), LPC (18:2), Indole-3-methyl acetate, and downregulating the levels of sphinganine 1-phosphate, L-threonic acid, glutaconic acid and N6,N6,N6-Trimethyl-L-lysine. In the research, sphinganine 1-phosphate decreased remarkably after the DSCXQ treatment both in ESI^+^ and ESI^–^ modes. Accordingly, we mainly concentrated on the sphingolipid metabolism pathway.

### DSCXQ Injection Ameliorate Neurological Scoring and Reduce Infarct Area in Cerebral Ischemia

**FIGURE 7:**
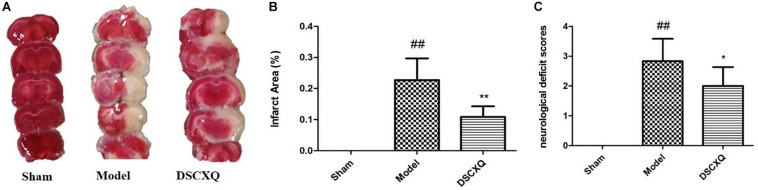
**(A)** Representative images of TTC staining in different groups of DSCXQ at 24h after I/R. Red areas represent normal tissue, while the white is infarction. **(B)** Quantitative analysis of infarct size in different groups of DSCXQ. **(C)** Effects of DSCXQ on neurologic deficits in different group, the evaluation of neurological deficits was assessed by four-point scale (0–4). Data were expressed with mean values ± standard deviation (SD). ##*p* < 0.01 vs. Sham group, **p < 0.01 *p < 0.05 vs. Model group.

The neurological damage was evaluated by an observer who was blinded to the experiments using a four-point scale, as described above. Neurological scores were shown in Figure 7C, and it was found that the neurological scores in DSCXQ group (score = 2) were lower compared with the Model group (score = 2.83), meaning the neurological symptoms were improved by the treated group. The results of TTC staining showed that the relative infarct area in the model group was increased significantly compared to sham group (Figure 7A), indicating our Model group was established successfully. With the treatment of DSCXQ, the area of cerebral infarction 10.86% was notably reduced in contrast with Model group 22.70% (Figure 7B, *p* < 0.01).

### Effects of DSCXQ on the Expression of SphK1, S1PR1 in Brain Tissues

**FIGURE 8:**
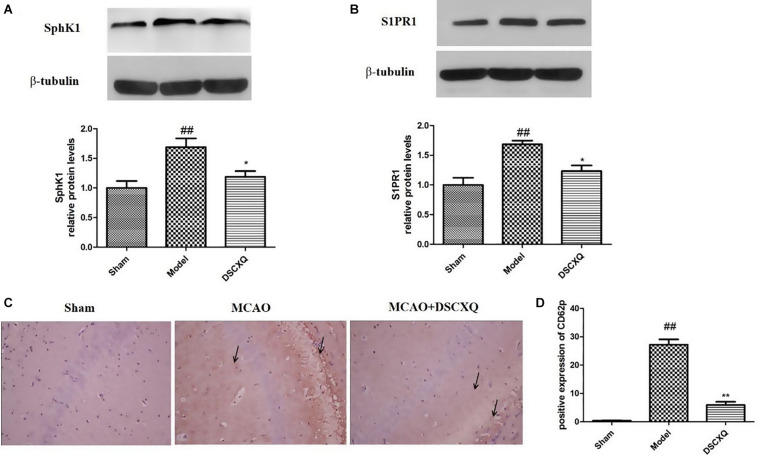
**(A,B)** Western blot analysis of Sphk1 and S1PR1 in the cerebral cortex and hippocampus of rats at 24h after MCAO. **(C,D)** Representative photographs represented the expression of CD62P in the cerebral cortex and hippocampus at 24h reperfusion after MCAO. The positive products were brown granules after immunostaining, which was remarkably increased followed MCAO and was significantly decreased after treatment with DSCXQ. Representative images of Sphk1 and S1PR1 were presented, and the protein levels were expressed as a ratio of the β-tubulin levels (*n* = 3). Data were expressed with mean values ± standard deviation (SD). ##*p* < 0.01 vs. Sham group, **p < 0.01 *p < 0.05 vs. Model group.

SphK1 is the dominant kinase for S1P production in the brain and exert a critical role in “sphingolipid rheostat.” S1P receptors, S1PR1 is amongst the most abundant subtype of S1P receptors in the brain, which plays a crucial role in sustaining hallmark endothelial functions. To validate the key enzymes and receptors in sphingomyelin metabolism, SphK1and S1PR1 were estimated by western blot (Figure 8). The results shows that expression levels of SphK1 and S1PR1 were increased notably following MCAO (*p* < 0.05), and compared to Model group, DSCXQ attenuated the upregulated SphK1 and increased levels S1PR1 (*p* < 0.05). These results verify that SphK1-mediated S1P production is increased in the injured brain after cerebral ischemia, and DSCXQ reduces the SphK1-induced S1P levels by suppressing S1PR1 receptors, which plays a crucial role in endothelial functions and vascular development.

### DSCXQ Reduced Immunoreactive Cells of CD62P

Platelets store large amounts of S1P that is only released upon activation (Nitzsche et al., 2021). CD62P, also known as P-selectin, can directly reflect the activation of platelets *in vivo* and interact with leukocytes to potentiate vascular injury. Immunohistochemistry was used to detect the expression of CD62P in cerebral cortex and hippocampus. In Figure 8C, there was little expression of CD62P in the sham operation group, however, the amount of CD62P in Model group was heavily activated, with the treatment of DSCXQ, the positive expression of CD62P decreased greatly compared with the Model group. The results, upregulation of CD62P + cells in Model group, further confirmed that the SphK1-S1P axis was activated, worsening the inflammatory response. And its downregulation by DSCXQ, indicating the neuroprotective effects of DSCXQ against neuroinflammatory responses.

### Effects of DSCXQ on the Expression of Bcl-2, Bax, Cleaved Caspase-3 in Brain Tissues

**FIGURE 9:**
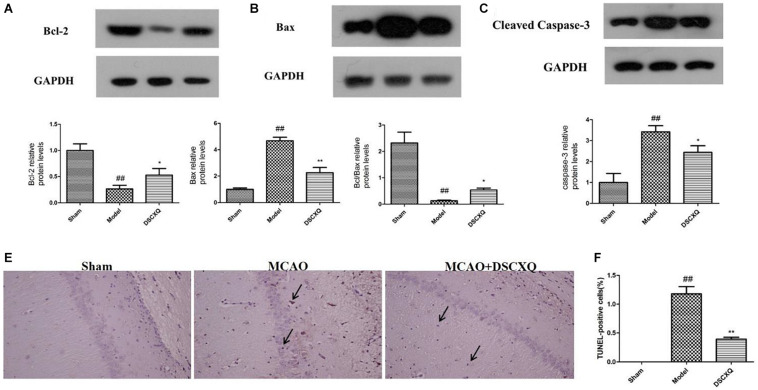
**(A-C)** Western blot analysis of Bcl-2, Bax, Cleaved Caspase-3 in the cerebral cortex and hippocampus of rats at 24h after MCAO. **(D,E)** Representative TUNEL-staining brain sections in different groups (*n* = 3) in the cerebral cortex and hippocampus at 24 h reperfusion after MCAO. Representative images of Bcl-2, Bax, Cleaved Caspase-3 protein were presented, and the protein levels were expressed as a ratio of the GAPDH levels (*n* = 3). Data were expressed with mean values ± standard deviation (SD). ##*p* < 0.01 vs. Sham group, **p < 0.01 *p < 0.05 vs. Model group.

SphK1/S1P axis has been involved in various physiological processes, including cell migration, survival, cell death, and it is a critical regulator of the sphingolipid rheostat (Maceyka et al., 2012). To further investigate the possible molecular mechanism of DSCXQ in stroke rats, the expression of Bcl-2, Bax, Cleaved Caspase-3 levels in brain tissue were determined by Western blot (Figure 9). The results revealed that the level of Bax and Cleaved Caspase-3 was up-regulated compared to Sham group (*p* < 0.05), while the content of Bcl-2 content in model group were down-regulated and the ratio of Bcl-2/Bax decreased notably compared with Sham group; With the treatment of DSCXQ, the result indicted that DSCXQ decreased the level of Bax and Cleaved Caspase-3, enhanced Bcl-2 greatly caused an increase in the ratio of Bcl-2/Bax significantly. The results, the remarkable downregulation of the ratio of Bcl-2/Bax in Model group, indicated that the balance between apoptosis and survival was broken and confirmed the SphK1-S1P axis was activated. With the treatment of DSCXQ, the downregulation of Bax, Cleaved Caspase-3, and up-regulated of Bcl-2, was achieved, implicating the neuroprotective effects of DSCXQ against neuronal apoptosis on a stroke model.

### DSCXQ Prevented Neuronal Apoptosis in Brain Tissues

As shown in Figure 9, in Model group, the positive cell shrinkage were brown granules after staining, which was remarkably increased compared with Sham group and was significantly decreased after treatment with DSCXQ. The results indicate that DSCXQ can effectively inhibit neuron apoptosis in cerebral ischemia.

## Discussion

In our study, neuroprotective effects of DSCXQ on ischemic stroke and the affected metabolic pathways were revealed by UHPLC-Q-Orbitrap HRMS-based metabolomics approach with multivariate statistical analysis methods. The results demonstrated that DSCXQ mediates its neuroprotective effect against stroke through modifying multiple metabolic pathways involved in lipid metabolism, amino metabolism, oxidative stress, and especially sphingolipid metabolism. our study clearly indicate that SphK1-SIP axis may potentiate neuroinflammatory responses and mediate brain damage in neuronal apoptosis, and DSCXQ suppressed the activity of SphK1-SIP axis to protect brain tissue in cerebral ischemia. Our work paved the way to uncovering the disturbed metabolic pathways, facilitating our understanding on neuroprotective mechanisms of DSCXQ against stroke from metabolomic insights.

### Sphingomyelin Metabolism and Apoptosis in Stroke

The metabolites of sphingolipids, such as ceramide, sphingosine and (dihydro)sphingosine-1-phosphate, are all signal lipid molecules and participate in many cell processes (Jin et al., 2003). A large number of documents show that Sph kinase (SphK), which phosphorylates Sph to form S1P, is a key regulator of the sphingolipid rheostat (Spiegel and Milstien, 2003), and SphK1 has been proved as the dominant kinase for S1P production in the brain and exert a critical role in “sphingolipid rheostat” (Maceyka et al., 2002; Blondeau et al., 2007). S1P receptors, S1PR1 is amongst the most abundant subtype of S1P receptors in the brain, which plays a crucial role in sustaining hallmark endothelial functions and could be as a regulator for microglial activation following cerebral ischemia (Moon et al., 2015; Nitzsche et al., 2021). S1P can activate platelets, which in turn stimulates the release of S1P into the bloodstream, S1P bind to receptors on the platelet surface, altering cell membrane glycoproteins, exposing fibrinogen receptors, increasing CD62P expression and activation rates and elevating platelet reactivity (Huang et al., 2006). CD62P, as an adhesion molecule on the surface of cell membrane, can mediate the mutual adhesion and aggregation of platelets, vascular endothelial cells, neutrophils and monocytes which play an important role in initiating thrombosis and exert a central role in inflammation and embolism. As platelets are activated, the CD62P is exposed to the surface of the cytosolic membrane and partially released into the blood. Hence, detecting the CD62P of platelet surface can directly reflect the activation degree of platelets in the body and understand the thrombosis process (Danton and Dietrich, 2003). Studies have shown that sphingosine is a lipid metabolite with multiple physiological and immunoregulatory functions, and may regulate apoptosis and necrosis (Radak et al., 2017). In our study, the significant increase of sphingomyelin and sphingosine caused abnormal sphingomyelin metabolism which up-regulated S1P expression, increased CD62P expression, intensified platelet activation, enhanced Bcl-2 level, inhibited cleaved Caspase-3 expression, induced cell necrosis and apoptosis - mechanisms which underlie the pathophysiology of stroke. CD62P immunohistochemistry revealed that number of CD62P^+^ cells was significantly high in Model group compared to that of Sham group, while the rats treated with DSCXQ showed a dramatic reduction in the expression of CD62P^+^ cells. To explore the possible molecular mechanisms related to beneficial effects of DSCXQ against apoptosis in I/R rats, the expression levels of Bax, Bcl-2 and cleaved Caspsae-3 in the rat brain were detected by western blot. The results indicate that the expressions of both Bax and cleaved Caspase-3 were markedly up-regulated and Bcl-2 markedly down-regulated compared with that of Sham group. However, DSCXQ administration, inhibited the expression of Bax and cleaved Caspase-3 expressions, while significantly enhanced the expression of Bcl-2 when compared with the non-treated Model group *(p* < 0.05). These results suggest that DSCXQ injection has significant anti-apoptotic effect against stroke.

### Ischemic Stroke and Amino Acid Metabolism

Compared with the Sham group, the amino acid metabolism in the MCAO group was obviously disordered, in which tryptophan and valine were negatively correlated with the occurrence of cerebral ischemia. Valine is one of branched amino acids (branched-chain amino acid, BACC) *in vivo*, which are closely related to neurotransmitter synthesis and protein degradation (Liu et al., 2016). Tryptophan, as one of the essential amino acids, plays an important role in maintaining the activity and increment of immune cells. On the other hand, studies have revealed that the active degradation of kyn pathway in Trp were closely linked to the severity and long-term prognosis of stroke (Cuartero et al., 2016), confirming our study.

Abnormal pathway of arginine metabolism may indicate the abnormal production of NO and the possible oxidative stress injury. As the central nervous system transmitter, the lack of NO may cause the brain information transmission disorder. At the early stage of cerebral ischemia, NO has the neuroprotective effect which can inhibit platelet and blood cell adhesion, researches have showed that NO in patients with acute cerebral infarction decreased significantly (Rashid et al., 2020), consistent with our findings. With the treatment of DSCXQ, the level of arginine was be on a downwards trend, indicating that the production of NO was likely to increase to exert neuroprotective effect.

### Lipid Metabolism and Stroke

Lipid metabolism disorder is an important factor causing stroke. Lipid compounds participate in cellular functions and energy storage, as well as a variety of precursor compounds as second messengers. Abnormal changes of lipid metabolism network in brain tissue may be closely related to changes of oxidative stress parameters reflecting apoptosis (Yang et al., 2017; Chen et al., 2019). In our study, the level of LysoPCs and LysoPEs decreased versus Sham group, among them, the level of LysoPE (18:0/0:0), LPC (18:2) increased after DSCXQ treatment which restored toward Sham groups.

Compared with the Sham group, the level of L-acetylcarnitine decreased in the cerebral ischemia model which played an important role in β-oxidation. Since brain is almost completely dependent on the oxidative energy supply of sugar. L-acetylcarnitine that is synthesized by acetyl CoA and carnitine mainly affects the energy metabolism of the brain (Zanelli et al., 2005). In the study, the decreased of 3-hydroxybutyrylcarnitine, the rise of propionylcarnitine, Butyrylcarnitine and 2-Methylbutyroylcarnitine were observed in Model group vs. Sham group, with the treatment of DSCXQ, the level of L-Acetylcarnitine, 2-Methylbutyroylcarnitine, 3-hydroxybutyrylcarnitine showed a trend toward Sham group, indicating that the neuroprotective effects of DSCXQ partially interferences with lipid metabolism in disease.

### Ischemic Stroke and Energy Metabolism

Energy metabolism disorder is the leading cause in the course of cerebral ischemia (Kurup et al., 1990). Cerebral ischemia restricts the delivery of oxygen and glucose in brain, and it has been revealed that energy metabolism shifted from tricarboxylic acid cycle to glycolysis. β-hydroxybutyrate, a small lipid-derived molecules which is classified as a type of ketone bodies, can be oxidized and provided energy when brain is in starvation, during glucose is insufficient, fatty acids were transported to liver where converted to ketone bodies, and transferred from blood serum to brain tissue (Newman and Verdin, 2017). In our experiments, β-hydroxybutyrate significantly decreased in MCAO rats and was raised by DSCXQ which may be alleviate energy supply obstacles and improve fatty acids utilization.

### Bile Acids –Gut Microbiome - Brain Axis in Stroke

Bile acids are the metabolites of cholesterol formed by a series of enzymatic reactions in hepatocytes. Meanwhile gut microbiome play a vital role in lipid regulation via promoting the formation of cholesterol oxidase. In our study, the primary bile acids in brain tissue of rats in Model group were significantly lower than those in Sham group. After the treatment of DSCXQ, the level of bile acids was notably higher than that in Model group. The lipids in the liver were cleared by bile acids, but it is interesting that bile acids in hepato-enteric circulation were detected in brain tissue, indicating that bile acid metabolism axis that runs through the liver - gut microbiota - brain is bound up with pathogenesis of stroke.

## Conclusion

In general, the research provides comprehensive insight into the mechanisms of DSCXQ against stroke by UHPLC-Q-Orbitrap HRMS based on metabolomics, and the results revealed that a series of complex and severe metabolic disorders were caused in MCAO rats after ischemic stroke injury. A total of 55 metabolic differences were detected as potential markers, and DSCXQ ameliorate the perturbed metabolic process mainly involving sphingolipid metabolism, amino acid metabolism, and cerebral homeostasis. Sphingolipid metabolism is closely associated with antiplatelet aggregation and cell survival. The results further verified that DSCXQ improved the cell survival and inhibited apoptosis in ischemia stroke rats. UHPLC-Q-Orbitrap HRMS-based metabolomics approach was applied in this study firstly to explore the neuroprotective effect of DSCXQ on ischemic stroke. By identifying the metabolic pathway and network, we can provide rich information to better understand and unravel the causal mechanisms of ischemic stroke, which has great potential in the future. According to the results of histopathology and metabolomics, the present study renovated our understanding of cerebral I/R injury pathogenesis and the neuroprotective mechanism of DSCXQ, which provided essential basis for clinical application.

## Data Availability Statement

The raw data supporting the conclusions of this article will be made available by the authors, without undue reservation.

## Ethics Statement

The animal study was reviewed and approved by the standard guidelines for the Care and Use of laboratory animals from the National Institute of Health (NIH) and the Animals Ethics Committee of Zheng Zhou University.

## Author Contributions

PZ contributed to the experimental design, implementation, and article writing. LZ participated in pharmacological experiments, contributed to data statistics, and contributed as a graphic analyst. YS, ZL, LL, and LHZ participated in experiments and differential metabolite identification. JZ participated in pharmacological experiments. JK, SL, JY, and SD partially participated in the experimental design. ZS designed the experiments and checked the project. XZ is the general director, project designer, and the teacher responsible for the funding, writing, and checking the project. All authors contributed to the article and approved the submitted version.

## Conflict of Interest

The authors declare that the research was conducted in the absence of any commercial or financial relationships that could be construed as a potential conflict of interest.

## Footnotes

1 https://www.kegg.jp/kegg/pathway.html

2 http://www.hmdb.ca/

## Abbreviations

DSCXQ: Danshen Chuanxinqin injection
MCAO: middle cerebral artery occlusion
I/R: ischemia reperfusion
Bcl-2: B-cell lymphoma-2
Bax: BCL2-associated X
MDA: malondialdehyde
t-PA: plasminogen activator
S1PR1: sphingosine 1-phosphate receptor-1
SphK1: sphingosine kinase 1
QC: quality control
TCM: traditional chinese medicine
CFDA: china food and drug administration.
