# Supplementary material for: Neuroprotective Effects of Danshen Chuanxiongqin Injection Against Ischemic Stroke: Metabolomic Insights by UHPLC-Q-Orbitrap HRMS Analysis
Source: Front Mol Biosci. 2021 May 7;8:630291. doi: 10.3389/fmolb.2021.630291 (PMC8138457; doi:10.3389/fmolb.2021.630291)
Supplement: Supplementary file 1 [file Table_1.docx]

1. Qualitative and quantitative analysis of Danshen-chuanxiong Injection

A qualitative analytical method of ultraperformance liquid chromatography-quadrupole/orbitrap high resolution mass spectrometry (UHPLC-Q-Orbitrap HRMS) was established for identification and quantification of the constituents of Danshen-Chuanxiong Injection. The method of UHPLC-HRMS was developed to identify the complex chemical composition and Full MS full scan mode was used to determinate the main active ingredient precisely, a total of 21 components had been identified, of which 12 compounds were confirmed by standards(Table 1) and 7 major compounds Tetramethylpyrazine, Salvianic aid A, Salvianolic acid A, Succinic Acid, Rosmarinic acid, 3,4-Dihydroxybenzaldehyde, Caffeic acid in Danshen-chuanxiong Injection were determined accurately(Table 2). The main bioactive compounds in DSCXQ were Ligustrazine and Danshensu.

(A)

(B)

Fig.1 Total ion current with positive (A) and negative (B) ion mode of Danshen Chuanxiongqin Injection

Table 1 Qualitative analysis information of Danshen-chuanxiong Injection

| No. | | Identification | Formula | Ion mode | | t_R/min_ | MS | | Error(ppm) | Fragment ions(m/z) |
| --- | --- | --- | --- | --- | --- | --- | --- | --- | --- | --- |
|  |  |  |  |  |  |  | Measured | Predicted |  |  |
| 1 | betaine | | C_5_H_11_NO_2_ | + | 1.63 | | 118.0865 | 118.0862 | 0.245 | 118.09, 59.07 |
| 2^*^ | Succinic acid | | C_4_H_6_O_4_ | - | 2.09 | | 117.0180 | 117.01933 | -0.265 | 99.01, 73.03 |
| 3 | 5-hydroxymethylfurfural | | C_6_H_6_O_3_ | + | 3.31 | | 127.0392 | 127.0390 | 0.219 | 109.03, 81.03, 53.04 |
| 4^*^ | Danshensu | | C_9_H_10_O_5_ | - | 3.49 | | 197.0455 | 197.0450 | -0.537 | 179.03, 151.04, 135.04, 123.04 |
| 5^*^ | Protocatechuic acid | | C_7_H_6_O_4_ | - | 4.13 | | 153.0182 | 153.0193 | -1.102 | 109.03, 91.02, 81.03 |
| 6^*^ | Ferulic acid | | C_10_H_10_O_4_ | + | 4.74 | | 195.0655 | 195.0652 | 0.275 | 177.05, 149.06, 131.05, 117.03, 103.05, 89.04 |
| 7^*^ | Rosmarinic acid | | C_18_H_16_O_8_ | - | 5.04 | | 359.0774 | 359.0772 | 0.416 | 197.04, 161.02, 133.03 |
| 8^*^ | Protocatechuic aldehyde | | C_7_H_6_O_3_ | - | 6.48 | | 137.0232 | 137.0244 | -1.237 | 119.01, 108.02, 91.02, 109.03 |
| 9^*^ | ligustrazine | | C_8_H_12_N_2_ | + | 8.30 | | 137.1073 | 137.1074 | 0.025 | 122.08, 107.06, 80.05, 209.12 |
| 10^*^ | caffeic acid | | C_9_H_8_O_4_ | - | 11.74 | | 179.0340 | 179.0349 | -0.952 | 135.04, 117.03, 107.05 |
| 11^*^ | Isoferulic acid | | C_10_H_10_O_4_ | + | 15.05 | | 195.0654 | 195.0652 | 0.998 | 177.05, 149.06, 131.05, 121.07, 103.05 |
| 12^*^ | Ligusticide D | | C_12_H_16_O_3_ | + | 16.77 | | 209.1182 | 209.1172 | 4.825 | 191.11, 167.07, 139.04, 135.04, 91.05 |
| 13 | Salvianolic acid H | | C_27_H_22_O_12_ | - | 19.30 | | 537.1041 | 537.1038 | 0.523 | 493.11, 295.06, 185.02, 109.03 |
| 14^*^ | Senkyunolide H | | C_12_H_16_O_4_ | + | 20.09 | | 225.1121 | 225.1121 | 0.02 | 207.10, 179.11, 165.09, 161.10 147.08, 137.10, 133.10, 119.09 |
| 15 | Salvianolic acid F | | C_17_H_14_O_6_ | - | 23.03 | | 313.0722 | 313.0718 | 1.305 | 269.08, 254.06, 237.06, 145.03 |
| 16 | Salvianolic acid E | | C_36_H_30_O_16_ | - | 24.59 | | 717.1453 | 717.1461 | -1.099 | 339.05, 321.04, 295.06, 185.02, 135.04, 109.03 |
| 17^*^ | Salvianolic acid A | | C_26_H_22_O_10_ | - | 24.87 | | 493.1135 | 493.1140 | -0.629 | 295.06, 185.02 |
| 18 | Salvianic acid C | | C_26_H_20_O_10_ | + | 24.88 | | 493.11252 | 493.11292 | -0.403 | 295.06, 267.07, 249.05, 221.06, 193.06, 165.07 |
| 19 | methyl rosmarinate | | C_19_H_18_O_8_ | - | 25.09 | | 373.0916 | 373.0929 | -3.352 | 179.03, 135.04 |
| 20 | ethy lithospermate | | C_29_H_26_O_12_ | - | 26.85 | | 565.1348 | 565.1351 | -0.565 | 367.08, 321.04, 293.05, 185.02, 135.04, 109.03 |
| 21 | Ethyl ferulate | | C_12_H_14_O_4_ | - | 31.30 | | 221.0815 | 221.0819 | -0.402 | 177.09, 149.10, 121.03, 71.05 |

Note：“*”means constituents identified with standards

Table 2 Determination of 7 main components in six batches

| Compounds | Mass Fraction /(μg∙ml^−1^) | | | | | | | |  |
| --- | --- | --- | --- | --- | --- | --- | --- | --- | --- |
|  | 1 | 2 | 3 | 4 | 5 | 6 | AVG±SD | | |
| Ligustrazine | 12572.35±143.77 | 12319.13±207.27 | 13002.70±190.63 | 11649.95±156.01 | 12241.83±168.07 | 12315.36±213.35 | | 12350.22±442.57 | |
| Danshensu | 502.89±8.39 | 481.13±9.00 | 483.75±8.90 | 490.89±8.01 | 522.92±6.63 | 498.77±7.62 | | 496.72±15.32 | |
| Salvianolic acid A | 68.87±0.57 | 59.19±0.91 | 71.80±0.53 | 119.35±1.40 | 128.21±1.29 | 54.83±0.78 | | 83.70±31.77 | |
| Succinic acid | 85.02±0.54 | 70.10±0.75 | 78.69±0.47 | 52.16±0.46 | 70.15±1.25 | 53.31±1.02 | | 68.24±13.26 | |
| Rosmarinic acid | 24.29±0.41 | 22.60±0.40 | 25.72±0.50 | 30.12±0.51 | 33.44±0.36 | 17.64±0.29 | | 25.63±5.58 | |
| Protocatechuic aldehyde | 8.44±0.15 | 12.10±0.14 | 11.82±0.18 | 11.63±0.16 | 20.73±0.13 | 11.11±0.09 | | 12.64±4.18 | |
| caffeic acid | 0.98±0.01 | 0.66±0.01 | 0.65±0.01 | 0.60±0.01 | 0.90±0.01 | 1.58±0.03 | | 0.89±0.37 | |

Note: the main bioactive compounds in DSCXQ are Ligustrazine and Danshensu. The level of Danshensu and Ligustrazine is an important index to control the consistency of quality standard of DSCXQ
